# Supplementary material for: Modeling the Repetition-Based Recovering of Acoustic and Visual Sources With Dendritic Neurons
Source: Front Neurosci. 2022 Apr 28;16:855753. doi: 10.3389/fnins.2022.855753 (PMC9097820; doi:10.3389/fnins.2022.855753)
Supplement: Supplementary file 1 [file Data_Sheet_1.PDF]

Supplementary Material

1 Supplementary Notes

1.1 Appendix: Methods

Below, we summarize the equations describing our two-compartment neuron model and synaptic learning rule. The details of the derivations were shown in the supplementary materials of the previous study (Asabuki and Fukai, 2020).

Two-compartment neuron model

The dendritic membrane potential of a two-compartment neuron obeys

$$v(t) = \sum_j w_j e_j(t), \qquad (1)$$

where w_j and e_j stand for the synaptic weight and the unit postsynaptic potential of the j-th presynaptic input. The somatic activity evolves as

$$\dot{u}(t) = -\frac{1}{\tau}u(t) + g_{\mathrm{D}}[-u(t) + v(t)] - \sum_j G_k \phi^{\mathrm{som}}\big(u_k(t)\big)/\phi_0, \quad (2)$$

where τ =15 ms and the conductance between the two compartments is $g_{\mathrm{D}} = 0.7$. The last term describes lateral inhibition with modifiable synaptic weights G_k (≥ 0), as shown later. The soma generates a Poisson spike train with the instantaneous firing rate $\phi^{\mathrm{som}}\big(u(t)\big)$, where

$$\phi^{\mathrm{som}}(u_i) = \phi_0\big[1 + \exp\big(\beta(-u + \theta)\big)\big]^{-1}, \qquad (3)$$

and the parameters β and θ are modified in an activity-dependent manner in terms of the mean $\mu(t)$ and variance $\sigma(t)$ of the membrane potential over a sufficiently long period t_0:

$$\beta = \sigma(t)^{-1}\beta_0, \qquad (4)$$

$$\theta = \mu(t) + \sigma(t)\theta_0, \qquad (5)$$

$$\mu(t) = \frac{1}{t_0}\int_{t-t_0}^{t} u(t')\,dt', \qquad (6)$$

$$\sigma(t) = \sqrt{\frac{1}{t_0}\int_{t-t_0}^{t} u(t')^2\,dt' - \mu(t)^2}. \qquad (7)$$

This online modification of the somatic response function maintains the dynamic range of output firing rate within a range adequate for learning. We set $\beta_0 = 5$ throughout this study, $\phi_0 = 1$ and $\theta_0 = 0.5$.

Sensory information given to the network is encoded into Poisson spike trains of input neuron $i \in \{1,2,\dots,N_{\text{in}}\}$ as

$$X_i(t) = \sum_q \delta\big(t - t_{i,q}\big), \qquad (8)$$

where δ is the Dirac' delta function and $t_{i,q}$ denotes the time of the q-th spike of neuron i. The presynaptic spikes induce the following synaptic current $I_i(t)$:

$$\tau_{\text{syn}} \dot{I}_i = -I_i + \frac{1}{\tau} X_i, \qquad (9)$$

where the synaptic time constant $\tau_{\text{syn}} = 5$ ms. The synaptic currents in turn evoke a postsynaptic potential $e_i(t)$ as

$$\dot{e}_i = -\frac{e_i}{\tau} + e_0 I_i, \qquad (10)$$

with the unit amplitude given as $e_0 = 25$.

Excitatory plasticity

To extract the repeated patterns from temporal input, the neuron model minimizes the following cost function, which represents the averaged KL-divergence between somatic activity and dendritic activity:

$$E(\mathbf{w}) = \int_{\Omega_{\mathbf{X}}} dX P^*(\mathbf{X}) \int_0^T dt \sum_i \mathrm{D_{KL}}\big[\phi_i^{\text{som}}\big(u_i(t;\mathbf{X})\big) \big\| \phi^{\text{dend}}\big(v_i^*(t;\mathbf{X})\big)\big], \quad (11)$$

with $P^*(\mathbf{X})$ and $\Omega_{\mathbf{X}}$ being the true distribution of input spike trains and the entire space spanned by them, and $\phi^{\text{dend}}(x) = \phi_0\big[1 + \exp\big(\beta_0(-x + \theta_0)\big)\big]^{-1}$. The sum runs over different neurons if multiple two-compartmental neurons exist in the network. Finally, minimizing the cost function and introducing the regularization term $-\gamma \mathbf{w}_i$ and a noise component ξ_i give the following learning rule:

$$\dot{\mathbf{w}}_i(t) = \eta \left\{\psi\big(v_i^*(t)\big)\big[\big\{f(\phi_i^{\text{som}} + \phi_0 g \xi_i) - \phi^{\text{dend}}\big(v_i^*(t)\big)\big\}/\phi_0\big]\mathbf{e}(t) - \gamma \mathbf{w}_i\right\}, \qquad (12)$$

where $\mathbf{w}_i = \big[w_{i1}, \cdots w_{iN_{\text{in}}}\big]$ and ξ_i obeys a normal distribution. The function $\psi(x)$ and f are defined as follows:

$$\psi(x) = \frac{d}{dx}\log\left(\phi^{\mathrm{dend}}(x)\right), \tag{13}$$

$$f(x) = \begin{cases} 0 & x < 0 \\ x & 0 \leq x < \phi_0 \\ \phi_0 & x \geq \phi_0 \end{cases}, \tag{14}$$

In Equation (12), the learning rate $\eta = 5 \cdot 10^{-6}$, and the strength of regularization and that of noise were set as $\gamma = 0.5$ and $g = 0.1$, respectively. Note that a smaller value was used for g compared to the previous model.

Inhibitory plasticity

If a pair of presynaptic and postsynaptic spikes occur at the times t_{pre} and t_{post}, respectively, lateral inhibitory connections between two-compartment neurons i and j were modified through a symmetric anti-Hebbian STDP as

$$\Delta G_{ij} = C_{\mathrm{p}} \exp\left(-\frac{|t_{\mathrm{pre}} - t_{\mathrm{post}}|}{\tau_{\mathrm{p}}}\right) - C_{\mathrm{d}} \exp\left(-\frac{|t_{\mathrm{pre}} - t_{\mathrm{post}}|}{\tau_{\mathrm{d}}}\right), \tag{15}$$

where $\tau_{\mathrm{p}} = 40$ ms, $\tau_{\mathrm{d}} = 20$ ms, $C_{\mathrm{p}} = 0.00525$ and $C_{\mathrm{d}} = 0.0105$. Inhibitory weights G_{ij} were modified between zero and an upper bound $G_{\max}$ ($\propto 1/\sqrt{N_{\mathrm{out}}}$).

2 Supplementary Figures and Tables

2.1 Supplementary Figures

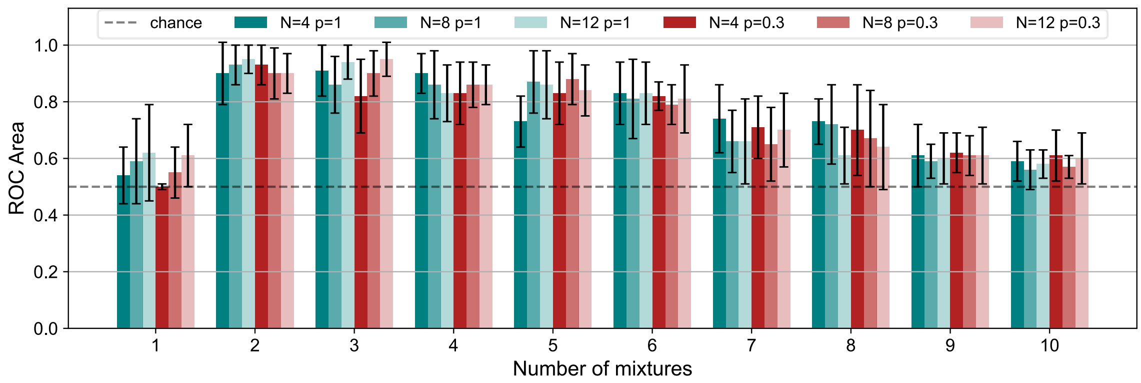

Supplementary Figure 1. Results for Experiment 1 run with different numbers of output neurons (N) and different connectivity probabilities (p).

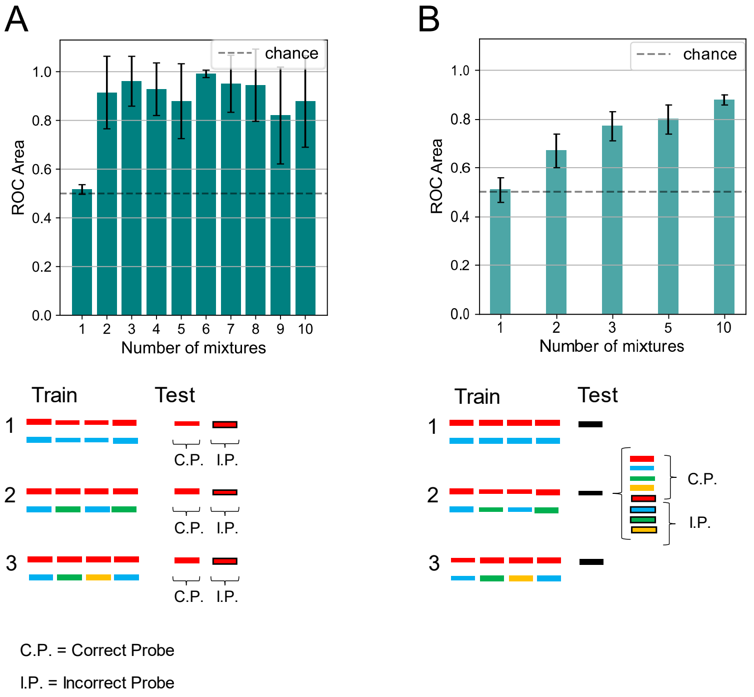

Supplementary Figure 2. (A) Results for Experiment 1 using during inference only the correct and incorrect probes related to the target sound. (B) Reference results from the human listeners' experiment.

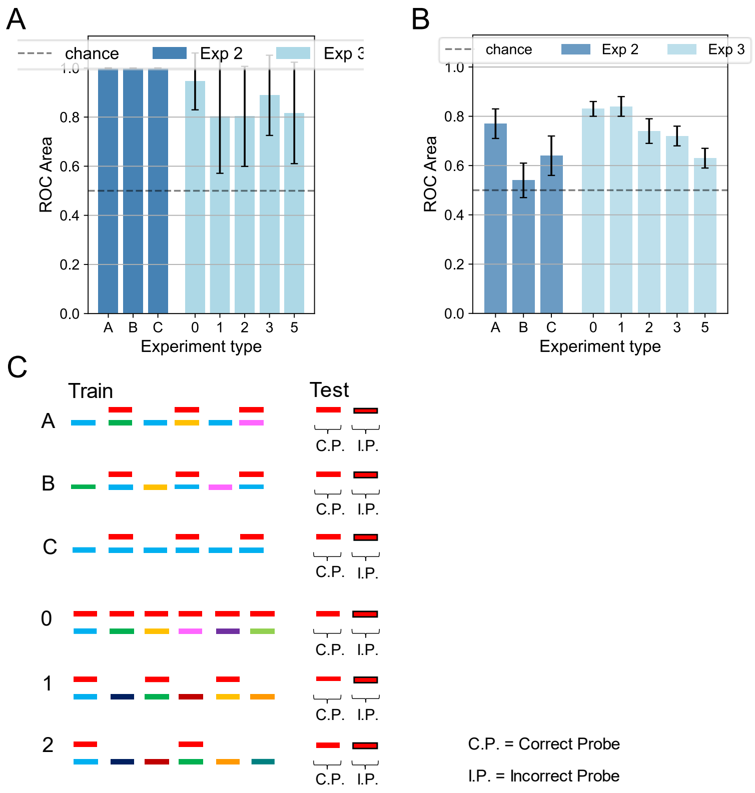

Supplementary Figure 3. (A) Results for Experiments 2 and 3 using during inference only the correct and incorrect probes related to the target sound. (B) Reference results from the human listeners' experiment.
